# Effectiveness of Virtual Reality-Based Interventions for Children and Adolescents with ADHD: A Systematic Review and Meta-Analysis

**DOI:** 10.3390/children8020070

**Published:** 2021-01-21

**Authors:** Dulce Romero-Ayuso, Abel Toledano-González, María del Carmen Rodríguez-Martínez, Palma Arroyo-Castillo, José Matías Triviño-Juárez, Pascual González, Patrocinio Ariza-Vega, Antonio Del Pino González, Antonio Segura-Fragoso

**Affiliations:** 1 Department of Physical Therapy, Occupational Therapy Division, Faculty of Health Sciences, University of Granada, 18016 Granada, Spain; palmarroyo@correo.ugr.es (P.A.-C.); pariza@ugr.es (P.A.-V.); 2 Faculty of Health Sciences, University of Castilla-La Mancha, Talavera la de Reina, 45600 Toledo, Spain; Abel.Toledano@uclm.es (A.T.-G.); Antonio.Segura@uclm.es (A.S.-F.); 3 Faculty of Health Sciences, Universidad de Málaga, 29016 Málaga, Spain; marrodmar@uma.es; 4 Primary Care Center Zaidín Sur, Granada Metropolitan Sanitary District, 18007 Granada, Spain; jmtjuarez@hotmail.com; 5 LoUISE Research Group, Computing Systems Department, University of Castilla-La Mancha, 02071 Albacete, Spain; Pascual.Gonzalez@uclm.es; 6 Department of Educational Orientation, IES Máximo Laguna, Consejería de Educación, Junta de Castilla-La Mancha, Santa Cruz de Mudela, 13730 Ciudad Real, Spain; antoniodelpino@hotmail.es

**Keywords:** virtual reality, ADHD, rehabilitation, cognition, attention, impulsivity

## Abstract

This review aims to evaluate the effectiveness of virtual reality-based interventions (VR-based interventions) on cognitive deficits in children with attention deficit hyperactivity disorder (ADHD). A systematic review and meta-analysis were performed according to the PRISMA statement and the Cochrane Handbook guidelines for conducting meta-analyses. The Grading of Recommendations, Assessment, Development and Evaluation (GRADE) was used to assess the quality of the evidence. Clinical trials published up to 29 October 2020, were included. The meta-analysis included four studies, with a population of 125 participants with ADHD. The magnitude of the effect was large for omissions (SMD = −1.38; *p* = 0.009), correct hits (SMD = −1.50; *p* = 0.004), and perceptual sensitivity (SMD = −1.07; *p* = 0.01); and moderate for commissions (SMD = −0.62; *p* = 0.002) and reaction time (SMD = −0.67; *p* = 0.03). The use of VR-based interventions for cognitive rehabilitation in children with ADHD is limited. The results showed that VR-based interventions are more effective in improving sustained attention. Improvements were observed in attentional vigilance measures, increasing the number of correct responses and decreasing the number of errors of omission. No improvements were observed in impulsivity responses.

## 1. Introduction

Among neurodevelopmental disorders, one of the most common is attention deficit hyperactivity disorder (ADHD) [1]. It is characterized by the presence of a persistent pattern of inattention and/or hyperactivity and impulsivity that interferes with cognitive functioning and participation in different activities, at least during the last six months. The diagnosis occurs after seven years of age and lasts throughout life [2]. Attention deficits are observed, for example, because the child frequently changes the focus of attention, particularly in monotonous and repetitive activities.

Occasionally, they do not pay attention to details, causing them to make mistakes or omit relevant information in the performance of tasks. It may also appear that they are not listening, even when people speak to them directly. They are easily distracted and forget details of activities of daily living (ADL) or lose objects. In addition, when impulsivity and hyperactivity appear, they can make decisions without reasoning, or not respect their turn in a conversation or play, generate difficulties in delaying gratification and inhibiting emotional reactions. This is mainly explained as a neurodevelopmental disorder of the prefrontal lobe, which concerns the development of executive functions in childhood. It includes inhibitory control, working memory, cognitive flexibility, planning, execution of goal-oriented behaviors, as well as self-behavior monitoring [3].

Some authors indicate that there is a long delay between the onset of symptoms and diagnosis and treatment, which affects the children’s functional performance, acquisition of skills, and development in a broad sense [4]. There are several systematic reviews of the use of technologies, both for assessment and intervention (serious games (SG), robots, virtual reality (VR), etc.) in children with neurodevelopmental disorders [1,4,5,6,7]. However, there are no reviews or meta-analyses on the effectiveness of virtual-reality interventions in children with ADHD. Previous studies suggest that it is necessary to perform specific research with a higher level of evidence of technological interventions [4,8]. Among the different technological systems, the potential of VR is highlighted. It began to be applied in the health field in the 1990s [9]. The differences between the applications of SG and VR systems is difficult, especially in the field of childhood, where the tasks usually consist of playing a game. Regardless of the game itself, there are aspects that clearly define VR: (1) the use of external tools that provide sensory information (mainly visual, auditory, and haptic) to interact with the virtual environment; (2) internal tools, which allow the collection of information on the user’s movement and position regarding their interaction with the system (for example, through gloves, trackers, exoskeletons, or a mouse); (3) systems for reproducing graphic images created by the virtual environment; and (4) software and databases that shape objects in the virtual world, using their shape, texture, or movement [8]. Furthermore, VR shares with the brain the mechanism of generating “embodied simulations” [8]. In fact, VR could be considered as an “embodied technology” [8] that provides the sensation of presence and immersion [10], allowing interaction, enhanced by the incorporation of virtual agents [11]. According to the hypothesis of predictive coding, it is understood that VR generates simulations of one’s own body in the world, through the developed scenario. It allows exploring and manipulating that environment, improving self-regulation and learning through the representation from the prediction of the internal (of the body itself) and external (environmental) sensory stimuli. These contribute to improving the movements, actions, and emotions adapted to the context [8,12]. Recent developments in augmented reality (AR), which allows the adding of information and the superimposing of information on the real world, facilitate the transfer of learning to everyday life, and achieve a high level of relevance and motivation [1]. In the case of children, both VR and AR have been considered positive technologies, since they improve the quality of experience, motivation, and learning [10]. They have the potential to allow the design of rehabilitation tasks focused on the child and according to the child’s interests, increasing their motivation in a safe environment and monitoring the their performance. These make VR a relevant tool in the field of rehabilitation.

In light of the above, the aim of this study was to evaluate the effectiveness of the use of VR-based interventions in children with ADHD, which is the most frequent neurodevelopmental disorder [4].

## 2. Materials and Methods

The systematic review was conducted according to the Cochrane Handbook for Systematic Reviews [8] (https://handbook-5-1.cochrane.org), and it was informed by the PRISMA Declaration guidelines [10]. The Grading of Recommendations, Assessment, Development and Evaluation (GRADE) system was used to assess the quality of the evidence [10]. This systematic review has been registered in PROSPERO under number code CRD42020152677.

### 2.1. Search Strategy

To assess the validity, applicability, and scope of this systematic review and meta-analysis, a search strategy was carried out in the Web of Science (WOS), Scopus, Cochrane and PsycINFO databases from 29 June to 29 October 2020. The terms included in the search are shown in Table 1. This string was applied to the title + abstract + keywords fields. The electronic search was completed with a review of the bibliographic references of the included studies.

### 2.2. Selection of Studies

Covidence (a web-based systematic review program that aims to make evidence synthesis a more proficient process, www.covidence.org) was used for the selection of articles. Randomized controlled clinical trials (RCT) or non-randomized controlled clinical trials that conducted VR-based interventions in children with a diagnosis of ADHD and that had been published in English or Spanish were included. Studies using immersive or semi-immersive VR or AR for the improvement of cognitive functioning were also selected. Articles (1) that did not include children or adolescents with ADHD; (2) that did not include VR-based interventions or AR, with robots or simple SG; (3) that were case series; (4) that were RCT protocols or clinical-trial protocols that did not present results; or (5) that focused on the application of VR exclusively to the educational field as a method of teaching a subject, were excluded.

No restrictions were placed on the minimum sample size of the studies or on the follow-up time. Two independent reviewers selected studies and extracted relevant data (A.T.-G. and D.R.-A.). Discrepancies were resolved by a third independent reviewer (M.C.R.-M.).

### 2.3. Data Extraction and Analysis

Four researchers (P.A.-C., A.T.-G., M.C.R.-M., D.R.-A.) carried out the bibliographic searches, and two reviewers (A.T.-G. and D.R.-A.) subsequently reviewed the relevance of the titles and abstracts according to the inclusion and exclusion criteria. Data extraction was performed by four researchers (P.A.-C., A.T.-G., M.C.R.-M., D.R.-A.). Clinical efficacy was defined by whether study results showed that a test or a treatment improved any symptoms [11]. A data-extraction form was developed that included the following data from each study: article title, country, year, author, type of study, sample size, follow-up time, main characteristics of the participants, description of the intervention, main outcomes, and conclusions.

### 2.4. Summary Measures

The objective measures of effect used to assess the effect of the intervention were Conners’ Continuous Performance Test (CPT) and the Virtual Classroom Task Assessment (VCTA). The CPT assesses sustained attention and vigilance for a simple task over a time interval [12]. It can be used in children aged four years to adulthood. The child must press a key when a letter appears on the computer screen, except when it is the letter “X”. The test lasts approximately 14 min. There are 324 non-X stimuli interspersed with 36 X stimuli, presented in blocks of trials with interstimulus intervals of 1, 2, and 4 s, which vary between blocks. Results for CPT performance include number of hits, number of omission errors, number of commission errors, hit reaction time (RT), perceptual sensitivity (d’), and response bias (B). The omissions refer to the lack of response to the target stimuli. Therefore, omissions indicate inattention, which can be motivated by a temporary lack of response or by looking away when the stimuli are presented. Commission errors, which refer to the responses given to stimuli other than the target stimulus, are caused by the inability to inhibit motor responses as a consequence of an impulsive response trend. Perceptual sensitivity (d’), which refers to errors due to difficulties in the discrimination of perceptual characteristics, is related to selective attention. Response bias (B) represents an individual’s response tendency.

The VCTA [13] is based on a continuous performance test and consists of a virtual system that uses a head-mounted display (HMD) that recreates a classroom with three rows of desks, a blackboard, and the teacher’s desk. One of the sides of the classroom is a window that overlooks a playground with buildings, vehicles, and people, while the other three sides are walls. The virtual teacher instructs the children to press the mouse when they see the letter “K” preceded by the letter “A”. There are auditory distractors (e.g., footsteps) visual distractors (e.g., a paper plane flying in the classroom), and mixed distractors (auditory and visual), like a car rumbling outside the window. The experiment consists of five blocks (over a period of 100 s each) with 20 targets (AK). Five hundred stimuli were presented throughout the task (500 s). The test registers the number of correct answers (number of cases in which an answer occurred together with target “AK”) and commission errors (number of cases in which a joint response occurred with a non-target).

### 2.5. Synthesis of Results (Statistical Analysis Plan)

The pre and post-intervention means with their standard deviations (SD) were used, and differences between scores after and before intervention (post-pre differences) were calculated. The SD not reported for these differences were calculated by imputing a correlation coefficient that was calculated in studies with the adequate information, from the pre- and post-SD and the SD of the difference. The weighted mean of these coefficients (*r* = 0.86) was calculated and applied to the rest of the studies. The effect size was determined using the adjusted Hedges G standardized mean difference (SMD) with a 95% confidence interval. The overall effect size was weighted by the sample size of the studies using the inverse variance method and a random-effects model. The 95% confidence interval (95% CI) and statistical significance were calculated using the z test. The magnitude of the effect was interpreted using Cohen’s criteria: 0.2: small effect; 0.5: medium; and 0.8: large effect. Satisfactory values ≥ 0.6 were considered [13]. The individual and the combined effect of all studies were plotted by forest plot using RevMan software [14], including assessment of risk of bias for individual studies. Due to the small sample size of the studies included in the meta-analysis, separate effects were calculated for each of the VCTA and CPT subscales: omissions, commissions, correct hits, reaction time, and perceptual sensitivity. All of these scales could not be combined into a single global estimate of the intervention effect.

### 2.6. Assessment of Risk of Bias in Individual Studies

An independent reviewer (A. S.-F.) assessed the methodological quality of the studies using the items included in RevMan: random sequence generation (selection bias), allocation concealment (selection bias), blinding of participants and personnel (performance bias), blinding of outcome assessment (detection bias), incomplete outcome data (attrition bias), and selective reporting (reporting bias). These items were categorized as ‘high’, ‘low’ or ‘unclear’.

### 2.7. Assessment of the Degree of Evidence of the Set of Studies

The GRADE system was used [9] to consider eight factors to assess the possible downgrade or upgrade of the level of quality of the evidence.

The GRADE system defines four levels of quality of evidence: high, moderate, low, and very low. The meta-analysis was based on controlled trials (randomized or non-randomized), so it was based on a high-quality level of evidence. Based on this, a series of factors were considered to downgrade or upgrade the final level of evidence.

The factors that downgraded the level of evidence were: (1) risk of bias of the set of studies; (2) inconsistency (heterogeneity) between studies; (3) indirect evidence; (4) imprecision; and (5) publication bias. The factors considered that upgraded the level of evidence were: (6) large effect magnitude (SMD > 0.8); (7) dose-response gradient; and (8) control for confounding factors.

#### 2.7.1. Risk of Bias of the Set of Studies

The studies that met the selection criteria were assessed with the items included in the review manager, according to the following characteristics: generation of the randomization sequence, concealment of the allocation sequence, blinding of participants and personnel, blinding of the outcome assessment, incomplete outcome data, and selective reporting of results. Each of these items was categorized as high, low, or unclear. Based on the joint assessment of the six items, the risk of bias of the study was categorized as follows: (1) low risk when all items were at low risk of bias; (2) unclear risk when one or more items have unclear risk; and (3) high risk when one or more items were at high risk of bias.

#### 2.7.2. Heterogeneity

To measure the degree of variability or heterogeneity in the effects of the intervention between the different studies, the I^2^ statistic (% of the variability of SMD attributable to heterogeneity and not to chance) was used, and the chi-square test was used to assess its statistical significance. It was assessed by visual examination of the forest plot and the chi-square statistic. The I^2^ was interpreted as absent (0%), low (25%), moderate (50%), or high heterogeneity (≥75%) [14]. The chi-square test was used to assess whether the differences observed between the studies were compatible with simple hazard [9].

#### 2.7.3. Indirect Evidence

Direct evidence is understood as that obtained from research that directly compares the interventions of interest, assessed in the type of patients in whom we are interested, and that measures relevant outcomes for patients. The level of evidence decreases when the population studied, the intervention, or the results measured are not adequate.

#### 2.7.4. Imprecision

Imprecision was assessed by the calculation of the optimal information size (OIS) [15], which is a conventional calculation to detect an SMD equal to the minimum clinically important difference and the post-SD of the control group. The calculation was performed using the comparison of the post-pre means in independent groups, based on the use of ANOVA of repeated measures (group-time interaction). Accepting an alpha risk of 0.05 and a beta risk of 0.1 (90% statistical power) in a two-sided contrast, the OIS would be 28 subjects in the experimental group and 28 in the control group to detect a standardized SMD effect size = 0.2.

#### 2.7.5. Publication Bias

Publication bias was assessed by visual examination of the funnel plot complemented with the DOI plot, both created with METAXL [16]. In addition, the Begg test and the Egger test calculated with Stata, and the Luis Furuya-Kanamori index (LFK) were used [17]. The Begg and Egger tests contrast the null hypothesis of absence of publication bias. Begg uses the rank correlation between the effect of the standardized intervention and its standard error [18]. Egger uses linear regression of the estimate of the intervention effect against its standard error, weighted by the inverse of the variance of the estimate of the intervention effect [19]. The LFK index uses a new graphical method, the DOI plot, to visualize the skew between studies. An LFK ≤ 1 was considered as no skewness = no publication bias; >1 ≤2 as minor skewness = low risk of publication bias; and > 2 as major skew = high risk of publication bias.

## 3. Results

### 3.1. Study Selection and Characteristics

**Figure 1:**
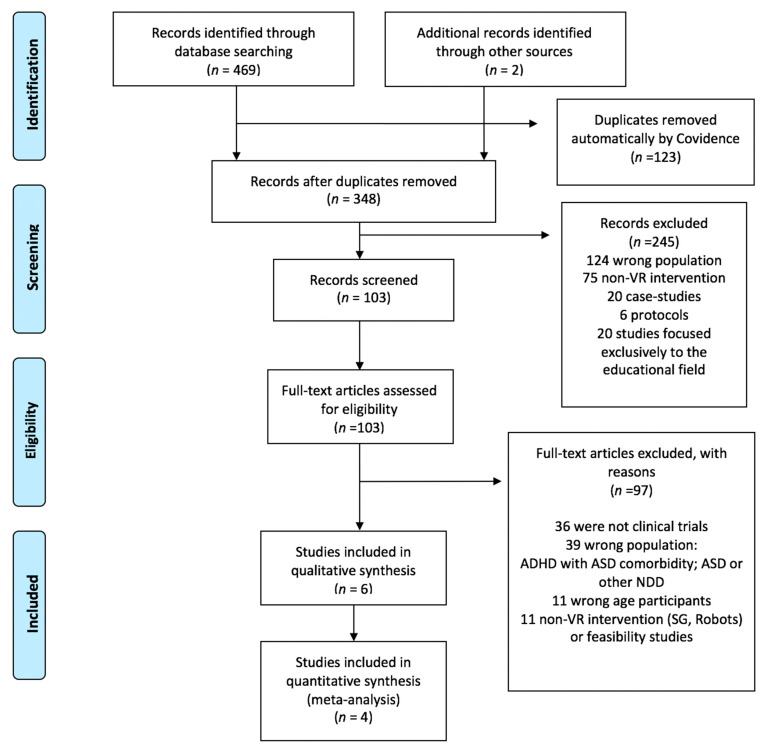
PRISMA flow diagram. ADHD: Attention deficit hyperactivity disorder; ASD: autism spectrum disorder; NDD: Neurodevelopmental disorder; VR: Virtual Reality; SG: Serious games.

A total of 471 studies were identified. Of these, 123 studies were duplicates and automatically discarded by Covidence. After de-duplicating, 348 manuscripts were reviewed by title and abstract, excluding 245 studies (124 included the wrong population; 75 were non-VR based interventions; 20 were case studies; 6 were protocols without results; and 20 were studies focused on the application of VR exclusively to the educational field), leaving 103 manuscripts for a complete reading. Of these 103 studies, 36 were not clinical trials; 39 included the wrong population (population with only autism spectrum disorder (ASD), ADHD with ASD comorbidity, or other neurodevelopmental disorders (NDD); 11 had a subject age > 18; and 11 used non-VR based interventions. The review of the full texts reported six studies. Of these, four were included in the meta-analysis (Figure 1; Table 2). The two studies not included in the meta-analysis were those of Tabrizi et al. [20] and Bul et al. [21]. Tabrizi et al. conducted a study in order to verify the effectiveness of virtual-reality systems to improve memory in children with ADHD. Eighteen children between seven and 12 years old participated in this study, divided into three groups: experimental, with pharmacological treatment, and control group. The type of sampling was intentional. The intervention consisted of 10 sessions. The virtual environment was a classroom in which different target stimuli appeared, and the child had to remember them at the end of the session. In addition, auditory and visual stimuli were incorporated, demanding greater inhibition of interference in the children. As the intervention advanced, the difficulty of the task also increased, increasing the number of targets and distractors. The results before and after the intervention were compared using the SNAP-4 questionnaire (a test of attention span and working memory (WISC-IV)) and the Raven Progressive Matrix test. After the intervention, the children who obtained the best results were those who followed VR. The results showed that VR-based interventions, similar to pharmacological treatment, improved memory in children with ADHD in both the post-test and follow-up stages. The study by Bul et al. [21] was the only one found that aimed to improve daily life skills, such as planning, time management, and social skills, through an SG named “Plan-It-Commander”, in which the child must perform 10 different missions. The sample was composed of 170 children diagnosed with ADHD according to the DSM-IV TR between eight and 12 years old, and of these, 88 of them received the intervention. The period of the intervention was 10 weeks, and it was used as a complementary treatment to a pharmacological one. To know the changes after the intervention (post-pre differences), a time-management questionnaire, the planning subscale of the Behavior Rating Inventory of Executive Function (BRIEF) questionnaire, and the cooperation subscale of the Social Skills Rating System (SSRS) were used [21]. According to the parents’ report, the children significantly improved their time-management skills (*p* = 0.04), social skills (*p* = 0.04), and working memory (*p* = 0.002), compared to the control group. The effects of the experimental group were maintained 10 weeks after the intervention.

### 3.2. Characteristics of the Studies Included in the Meta-Analysis

The four studies finally included in the meta-analysis are summarized in Table 2. Of these, three were RCT [22,23,24] and one was a non-randomized controlled trial [25]. The type of experimental intervention was immersive VR using a head-mounted display (HMD), and in some studies using a head tracker with 3 degrees of freedom (DOF) [25]. The study presented by Lee et al. [25] also performed EEG recordings, and in Cho’s study [22], neurofeedback was used at the same time as VR, although the neurofeedback protocol was not reported. The total number of participants included in the meta-analysis was 125; of these, 44 were in the experimental group and 81 were in the comparison one. The mean age was 12.9 years. The vast majority were male (92%).

The clinical profile of the participants was characterized by presenting the clinical symptoms for the diagnosis of ADHD according to the DSM-IV. Three of the four studies indicated that they had no previous experience with the use of VR-based interventions, and that all the participants did so voluntarily and could withdraw at any time from the study.

### 3.3. Interventions

All VR-based interventions used HMD systems with immersive, through cognitive tasks of attention, where the child was instructed to keep their attention focused on the activity, to select targets, and to inhibit responses to target stimuli. In all the studies, the interventions were individual. In Lee’s study [25], the setting was a game with a dinosaur. In the other three studies, the virtual environment simulated a classroom. The type of control interventions in the case of the study by Lee et al. [25] received no intervention. In the study by Bioulac et al. [23], there were two comparison groups, one of which received only pharmacological treatment with QUASYM, and the other of which received only supportive psychotherapy and psychoeducation. In the two studies by Cho et al. [22,24], there were two comparison groups, one of which received cognitive rehabilitation through computerized tasks, while the control group received no treatment. The intervention period in three of the four studies was two weeks. Only the most recent study by Biolac et al. [23] lasted six weeks. The number of sessions varied from eight to 12 (Table 2).

### 3.4. Effect of the VR-Based Interventions on the Different Factors of Sustained Attention and Impulsivity in Children with ADHD

**Figure 2:**
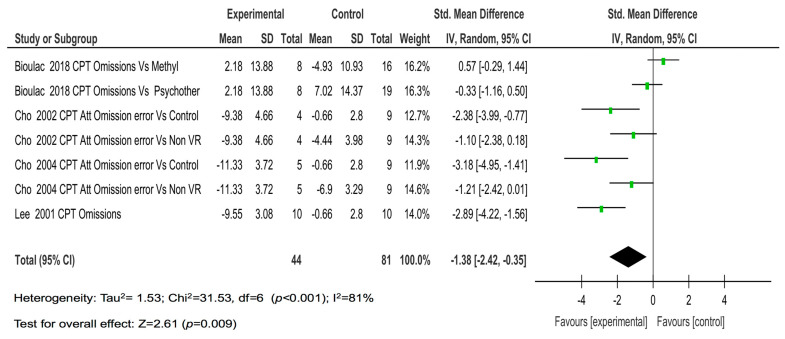
The results of the individual studies and meta-analysis on omissions.

Figure 2 shows the forest plot with the results of the individual studies and the meta-analysis on omissions. In summary, the four studies with seven comparisons showed a large magnitude of the effect on omissions (SMD = −1.38; *p* = 0.009).

**Figure 3:**
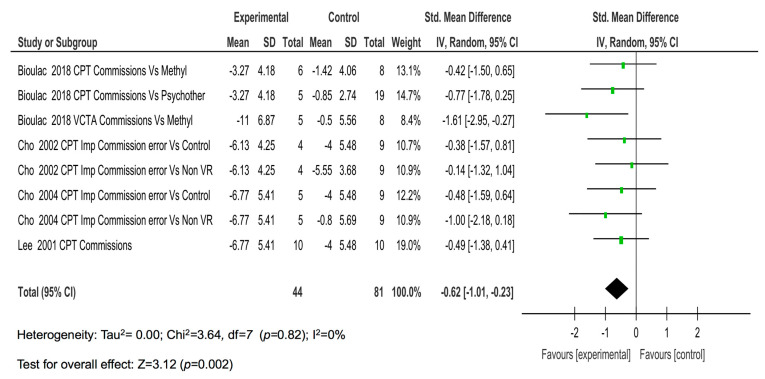
The results of the individual studies and meta-analysis on commissions.

The four studies with eight comparisons showed a moderate magnitude of the effect on commissions (SMD = −0.62; *p* = 0.002) (Figure 3).

**Figure 4:**
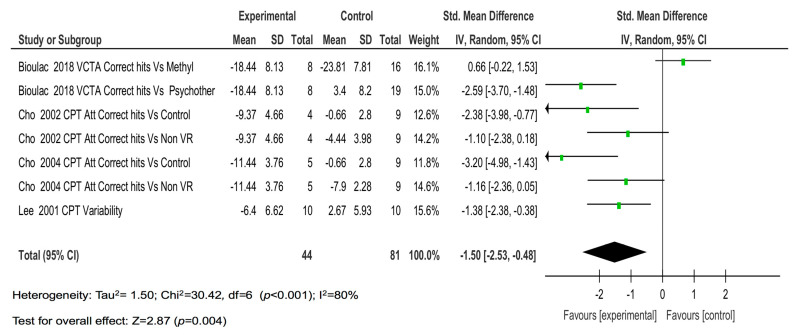
The results of the individual studies and meta-analysis on correct hits.

The four studies with seven comparisons showed a large magnitude of the effect for correct hits (SMD = −1.50; *p* = 0.004) (Figure 4).

**Figure 5:**
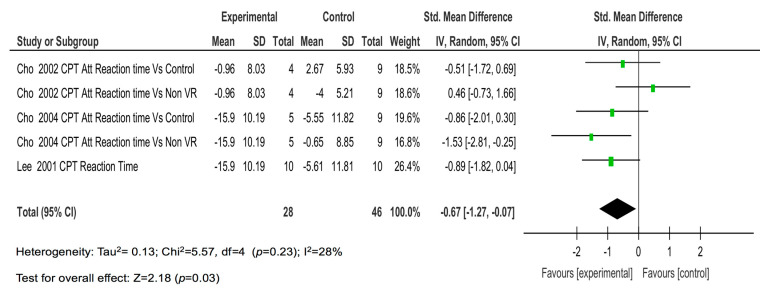
The results of the individual studies and meta-analysis on reaction time.

**Figure 6:**
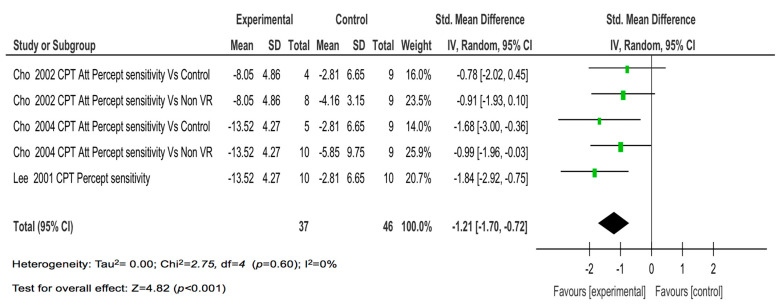
The results of the individual studies and meta-analysis on perceptual sensitivity.

Finally, the four studies with five comparisons on the reaction time showed a moderate magnitude of the effect (SMD = −0.67; *p* = 0.03) (Figure 5) and a large magnitude of the effect on perceptual sensitivity (SMD = −1.21; *p* <0.001) (Figure 6).

### 3.5. Degree of Evidence from the Set of Studies

#### 3.5.1. Risk of Bias of Individual Studies

**Figure 7:**
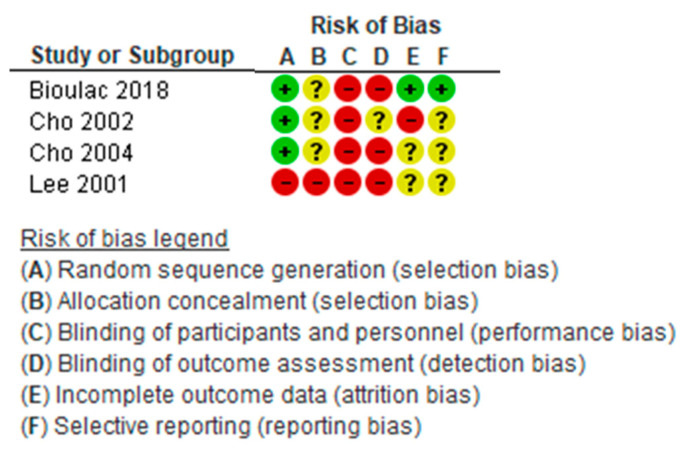
The risk of bias in the individual studies. green = low risk of bias; yellow with question mark = unclear risk of bias; red = high risk of bias.

Figure 7 shows the risk of bias for each study in each of the assessed criteria, using three colors: green = low risk; yellow with question mark = unclear; and red = high risk. All studies except the one by Lee et al. showed a low risk in the generation of the randomization sequence; three of the studies showed an unclear risk of bias in the allocation concealment, while in the study of Lee et al., this risk was high. All were at high risk of bias in the blinding of participants and personnel, and three of them in the blinding of outcome assessment. Only one study had a low risk of bias in incomplete outcome data and in selective reporting.

#### 3.5.2. Heterogeneity

Total heterogeneity was high in omissions (I² = 81%; *p* < 0.009) and correct hits (I² = 80%; *p* < 0.004). It was moderate in perceptual sensitivity (I² = 59%; *p* < 0.04), low in reaction time (I² = 28%; *p* < 0.23), and null in commissions (I² = 0%; *p* < 0.82). This indicated inconsistency in the results for omissions, correct hits, and perceptual sensitivity.

#### 3.5.3. Indirect Evidence

None of the studies found substantial differences among the study population, the intervention or the outcomes measured, and the criteria established in the meta-analysis.

#### 3.5.4. Imprecision

The total sample size of the studies included in the meta-analysis was 125 for omissions, commissions, and correct hits (44 in the experimental group and 81 in the control group), and 74 for reaction time and perceptual sensitivity (28 in the experimental group and 46 in the control group).

#### 3.5.5. Publication Bias

**Figure 8:**
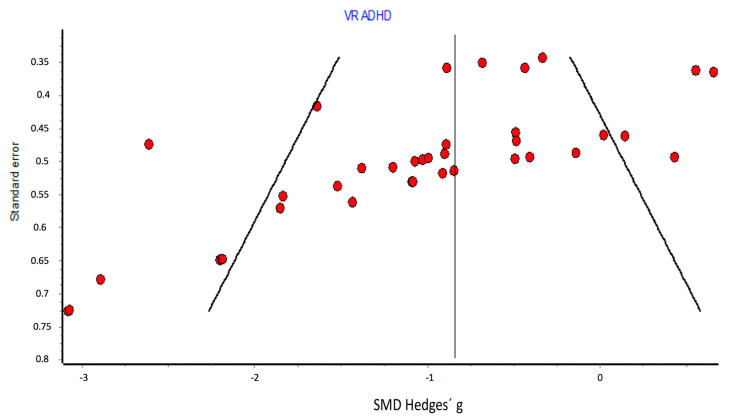
Publication bias: funnel plot.

**Figure 9:**
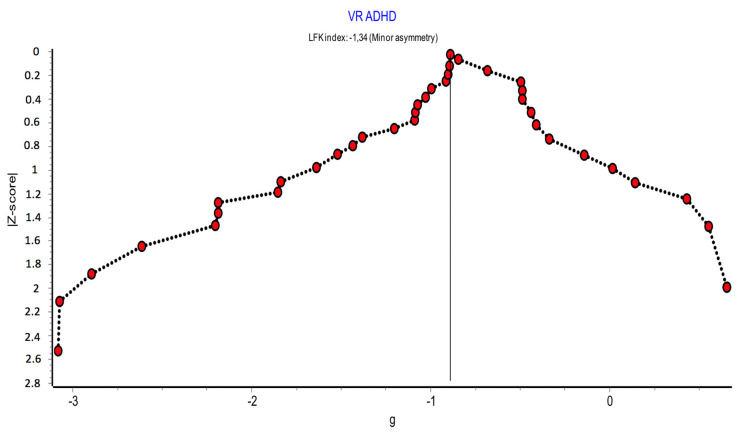
Publication bias: DOI plot and LFK index.

Figure 8 and Figure 9 show the assessment of publication bias using the funnel plot and DOI plot.

There was a certain asymmetry between the comparisons whose SMD was below or above the mean of all comparisons. Comparisons with larger effects (left of the plot) also had smaller sample sizes (larger standard errors), while those with smaller SMDs (right of the plot) had smaller standard errors and therefore larger sample sizes. It could be considered that there is a deficit of results in the lower-right quadrant of the plot. It would be a consistent bias in the non-publication of small studies with little or no effects.

There was a certain asymmetry similar to that described in the funnel plot. The LFK index showed minor asymmetry (LFK = −1.34). The statistical significance of the Begg test was *p* < 0.001, and that of the Egger test was *p* < 0.001.

## 4. Discussion

The aim of this meta-analysis was to know the effectiveness of VR-based interventions in ADHD children and adolescents. According to Ioannidis [26], the present study tries to overcome the drawbacks that many meta-analyses have. Therefore, our research sought to promote evidence-based practice. Our meta-analysis showed that there are few studies of VR-based interventions aimed at the cognitive rehabilitation of ADHD children. Most VR studies in ADHD populations have focused on validating the assessment of attention in a virtual classroom environment [4,13,27,28,29]. Furthermore, as in other NDD [30], there is a lack of consensus on the outcome measures used in the different studies, which means that there are few studies that can be compared. However, in our review, we have only included studies with the same outcome measures. In this sense, this represents a strength of our meta-analysis, since this is a consequence of establishing rigorous criteria on the type of outcome measures and type of intervention, with the aim of knowing more clearly the effectiveness of VR-based interventions, and considering the difficulties noted by other meta-analysis that have addressed the effectiveness of psychosocial treatment in ADHD [31]. Moreover, our study attempted to respond to the criticism made by another meta-analysis of meta-analysis of psychosocial treatments in ADHD, in which the authors address this issue as the problem of: *Apples and Apples or Apples and Oranges*? [31].

All the studies except for Lee’s [25] used a classroom as a virtual environment and focused on cognitive tasks, similar to those required by the CPT-type sustained attention tests. In this way, there was a notable reduction in the type of demands for vigilance and sustained attention that have been raised in the studies. It is noticeable that this is so when, in addition to attention deficits, children with ADHD have significant executive deficits, such as planning, execution, and supervision of action, which requires the development of self-regulation. In addition, the deficits of these children also affect the scope of activities of daily living, social and recreational activities. Only the study by Bul et al. [21] attempted to address this type of difficulties, although it did so using SG. On the other hand, the study by Tabrizi only tried to address the deficits in working memory in these children, but again in the virtual classroom setting [20].

### 4.1. Effect of VR-Based Interventions on Each Type of Outcome (Omissions, Commissions, Correct Hits, Reaction Time, Perceptual Sensitivity)

In summary, although there were a limited number of studies, the results suggest that VR-based interventions help to improve the cognitive performance of children and adolescents with ADHD in vigilance and sustained-attention tasks, reducing the number of omissions, and increasing the number of correct responses to the target stimuli with large effect size. Meanwhile, a medium effect on performance was observed in the reaction time to the target stimuli and the number of errors per commission. This suggests that the effect is more notable on vigilance, and less on the improvement of impulsivity or control inhibitory. These results are of interest because they suggest that VR-based interventions could improve inattention symptoms and therefore be very useful in children with ADHD of the inattention subtype, in whom a greater number of omissions and fewer correct answers have been observed [1].

On the other hand, the results of the Bioulac study [23] showed that children who received VR-based interventions can better inhibit distractors. Moreover, they also showed less impulsivity (with a lower number of commissions). The authors of this study also indicated the good acceptability of this type of intervention. However, no improvements were observed by parents according to the results of the ADHD-RS, showing that although there was an improvement in the parameters of the attention tasks, there was no transfer to activities of daily living [32], which suggests that it is necessary to increase the ecological validity of this type of intervention, as mentioned by the authors themselves. The simple fact of modifying the environment of the task does not mean that the task has ecological validity (pressing a button when a stimulus appears). It is necessary to differentiate between tasks and environment and carefully analyze the task and adapt it so it has ecological validity in accordance with age-appropriate demands, and with significant value for the child [33].

### 4.2. GRADE Quality of Evidence

#### 4.2.1. Assessment of Risk of Bias in the Individual Studies

Risk of bias is considered high, as all studies had one or more items with a high risk of bias. For this reason, it is considered to downgrade the level of evidence in omissions, commissions, correct hits, reaction time, and perceptual sensitivity.

#### 4.2.2. Assessment of Heterogeneity

In omissions and correct hits, evidence was downgraded a level for heterogeneity because the studies presented a high heterogeneity. Downgrading was not considered for commissions, reaction time, and perceptual sensitivity.

#### 4.2.3. Assessment of Indirect Measurement

It was not considered to downgrade the level of evidence by indirect measurements, since all measurements were direct.

#### 4.2.4. Assessment of Imprecision

Because the sample size equaled or exceeded the OIS in all outcomes, it was not considered to downgrade one level due to imprecision.

#### 4.2.5. Assessment of Publication Bias

Despite the fact that there was a certain degree of asymmetry and risk of publication bias, and the statistical significance of the Begg and Egger tests lead to rejecting the null hypothesis of the absence of publication bias, the LFK index showed less asymmetry (LFK = −1.34). Therefore, it was not considered to downgrade a level due to publication bias.

The criteria used to upgrade the level of evidence were: (1) Large effect size—it was considered to upgrade one level in omissions and correct hits. It was not considered to upgrade a level of evidence in commissions, reaction time, and perceptual sensitivity, since the observed effect showed a moderate or small improvement; (2) Dose-response effect—it was not considered to upgrade the level of evidence because the dose-response effect was not assessable in this meta-analysis (there was not a gradual exposure to the intervention in any of the studies included); and (3) Control of blinding factors—although it was considered that the control of blinding was relatively good in the case of the three RCTs and the non-randomized controlled trial, it was not considered to upgrade a level of evidence, since most of the studies had small sample sizes. Therefore, in summary, the GRADE level of evidence on the effects of VR-based interventions remains as seen in Table 3.

This study has a number of limitations. First, it is likely that not all studies were identified, despite using extensive search strategies. Second, the variety of clinical settings, evaluation protocols, and interventions did not allow the findings to be generalized. Third, the methodological shortcomings of the studies and the absence of post-intervention follow-up did not allow us to know if the effect achieved is lasting. Fourth, the studies included have small sample sizes. Meta-analyses based on studies with small sample sizes could produce heterogeneous effect sizes. In this meta-analysis, heterogeneity was high in some results, indicating an inconsistency in the findings of the various studies, so they should be interpreted with caution.

Although the number of studies with VR-based interventions as a complementary therapy in ADHD has increased, the vast majority are intra-subject design research [17], without a comparison group [17,18,19] or cross-sectional studies conducted to find the specific performance in a virtual task [20,21]. The results of our study show the need for more robust RCTs with larger sample sizes and with methodological planning that includes blinding of participants and personnel, blinding of outcome assessments, and allocation concealment. As stated in a recent systematic review, there is a limited number of RCTs; studies on VR-based interventions are scarce, mostly without control groups and with small sample sizes [11], and have very different outcomes. It is necessary to encourage studies that include VR-based interventions with higher methodological quality that allow conclusions to be drawn and provide evidence of their effectiveness.

Given the advances in VR systems, which allow simulating the body and interoceptive and proprioceptive perception [34], future studies could improve self-regulation in children with ADHD in different contexts of daily life, and it is recommended that the tasks implemented through VR have ecological validity [33]. Furthermore, it is necessary to develop more studies that address the planning and supervision of the action and organizational skills, since they are symptoms that continue into adulthood and are hardly addressed in current approaches [35], being basic skills for personal autonomy in ADL [33,36]. Likewise, other studies have indicated the need for the technologies used with children with NDD to be flexible and adaptable, given the heterogeneity of the needs of each child [4,37]. Finally, we note that VR-based interventions, in children between six and 11 years old, could make it difficult to for them to distinguish between memories based on VR and real ones [13]; therefore, the use of VR-based interventions should always be supervised by a healthcare professional [9]. In the future, we consider studies in which brain activity is recorded simultaneously with the use of VR-based interventions to be of interest.

## 5. Conclusions

To the best of our knowledge, this is the first systematic review and meta-analysis on the effectiveness of VR-based interventions in children and adolescents with ADHD. The studies included in this meta-analysis were three RCTs and one non-randomized controlled trial that analyzed the effect of VR-based interventions to improve cognitive functioning in children with ADHD and that were published between 2001 and 2020.

The review analyzed 469 articles. We selected four studies that included immersive or semi-immersive VR-based interventions [22,23,24,25]. No RCTs or non-randomized controlled trials with augmented or semi-immersive reality were found. The total number of participants among all the studies included was 125 children and adolescents with ADHD (115 males and 10 females); the ages ranged from eight to 18 years, with a mean age of 12.9 years.

Our study provides relevant results for scientific advancement in the design and implementation of new VR-based interventions. VR-based interventions were effective in improving cognitive performance in ADHD, such as sustained vigilance, which showed a decrease in omissions.

Future RCTs of VR-based interventions should consider the following recommendations: (1) New studies should include other virtual environments alternative to the classroom, such as free-play environments (for example, a school playground, park, etc.), basic activities of daily living, environments where the main demand is social, or the use of the environment, such as that of the instrumental activities of daily living; (2) New interventions should include tasks that require the child to plan and supervise action, adherence to rules, correction of errors, and working memory, because these are core deficits in ADHD; (3) In addition, it would be convenient for the new RCTs to include different ADHD comparison groups, especially due to its prevalence in children with only inattentive subtypes and with hyperactive-impulsive predominance (to date there are none); (4) The new VR-based interventions should allow graduating these cognitive and social demands according to the age of the child and the deficits severity; and (5)new studies should include follow-up measures to determine if the improvement is maintained over time. All of these recommendations will help researchers and clinicians to design studies and tools with greater ecological validity.

## Figures and Tables

**Table 1:** Key Search Terms.

	Key Seach Terms
Population	(ADHD OR attention deficit OR hyperactivity disorder) AND children
Intervention	virtual reality OR virtual environment OR virtual rehabilitation OR augmented reality OR serious games
Comparation	neurorehabilitation OR cognitive training OR cognitive therapy OR neuropsychological rehabilitation OR neuropsychological training OR neuropsychological therapy OR attention training
Outcome	cognition OR attention OR sustained attention OR impulsivity OR cognitive impulsivity OR executive function

**Table 2:** The studies included in the meta-analysis.

Authors and Year	Title	Measures	Intervention: Experimental Group	Intervention: Comparison Group	**Outcome/Results**
Bioulac, S., Micoulad-Franchi, J.A., Maire, J., Bouvard, M.P., Rizzo, A.A., Sagaspe, P. & Philip, P. 2020 [23]	Virtual Remediation versus methylphenidate to improve distractibility in children with ADHD: A controlled randomized clinical trial study	ADHD-RS Symptom Inventory according to DSM-IV Continuous Performance Test (CPT) Virtual AULA (HMD)	The experimental group received VR-based intervention. No. of participants = 16 children with ADHD, between 7 and 11 years old, with Diagnosis according to DSM-IV; with an IQ > 85 and a Score > 28 on the ADHD-RS. No. of sessions = 12 Duration of sessions = 30 min, Frequency of sessions = 2 times a week Intervention period = 6 weeks Children were asked to detect letters while inhibiting various distractors (e.g., falling pencils, footsteps, intercom announcements, etc.)	**Group with long-acting methylphenidate (QUASYM) ** No. of participants = 16 A clinical interview was conducted every two weeks for 8 weeks. The maximum dosage was 1 mg/Kg. **Group with placebo psychotherapy training** No. of participants = 16 Duration of sessions = 30 min Intervention period = 6–8 weeks, No. of sessions = 12 The intervention focused on attention, support and psychoeducation.	The children who received the VR-based intervention obtained higher performance in the tasks and tests of sustained attention both in the CPT and in the virtual AULA tests. After intervention, there were no differences in the number of omissions in the CPT between the VR-based intervention group and methylphenidate group (*p* = 0.03). There were differences due to commission errors between these two groups, being lower in VR-based intervention group (*p* = 0.001); The number of hits in the virtual classroom cognitive remediation group was significantly higher than in the psychotherapy group (*p* < 0.001). There were no differences in the number of commissions between the groups with psychotherapy and pharmacological treatment. In the CPT task, there were significant differences in the number of commissions between the virtual classroom cognitive rehabilitation group and the methylphenidate group (*p* = 0.05). No child in the VR-based intervention group reported adverse effects, such as cybersickness.
Cho, et al., 2002 [24]	The Effect of Virtual Reality Cognitive Training for Attention Enhancement	Continuous Performance Test (CPT)	**VR-based intervention group (HMD)** No. of participants = 9 No. of sessions = 8 Duration of sessions = 20 min Intervention period = 2 weeks Treatment focuses on tasks of sustained, selective, divided or alternating attention, with different tasks where they have to stop an activity at a signal, as a flag, comparing objects and observe similarities, etc.	**Group Non-VR** No. of participants = 9 Cognitive rehabilitation similar to VR but using a computer **Control group** No. of participants = 9 They received no intervention	Perceptual sensitivity decreased in all groups, being higher and significant in the VR-based intervention group (*p* < 0.01). Only the response bias decreased in the VR-based intervention group (*p* < 0.01). Correct hits on the CPT was higher in the VR-based intervention group, although the differences were not statistically significant.
Cho et al., 2004 [22]	Neurofeedback training with VR for inattention and impulsiveness	Continuous Performance Test CPT	**VR-based intervention group (HMD)** VE was a classroom No. of participants = 10 No. of sessions = 8 Duration of sessions = 20 min Intervention period = 2 weeks	**Non-VR Group = 9** Cognitive rehabilitation similar to VR but using a computer. **Control Group = 9** They received no intervention.	The VR-based intervention group improved the number of correct responses after the intervention (*p* < 0.001), decreased its reaction time (*p* < 0.001), the perceptual sensitivity (*p* < 0.01) and commissions (*p* < 0.05) after the intervention. Response bias increased in all groups (*p* < 0.001). The group that improved the most in the impulsivity parameters was the VR-based intervention one.
Lee et al., 2001 [25]	A study on the system for treatment of ADHD using virtual reality	Continuous Performance Test (CPT)	**VR-based intervention group** HDM and tracking system with three EEG electrodes (Cz, grounded in the right and left ears). The EEG signal acquisition frequency was 256 Hz. They extracted the frequency parameters Delta (0.5–3 Hz), Theta (4–7 Hz), Alpha (8–12 Hz), SMR (12–15 Hz) and Beta (15–18 Hz). The data was updated every 0.5 s. No. of participants = 10 No. of sessions = 10 Duration of each session = 10 min Intervention period = 2 weeks When the child’s Beta threshold value increased (15–18 Hz), there was a small change in the virtual environment. The virtual task was centered on dinosaurs, where information about them was presented and then the child was asked to answer a series of questions about the information presented.	**Control Group** No. of participants = 10 They received no intervention	The VR-based intervention group showed a reduction of omissions and commissions versus the control group. The perceptual sensitivity decreased after VR-based intervention

**Table 3:** The assessment of the quality of the studies.

							Global Effect	
	No. Studies (Comparisons)	Risk of Bias	Inconsistency Heterogeneity	Indirect Evidence	Imprecision	Publication Bias	SMD (CI 95%)	Quality of Evidence
Omissions	4 (7)	High (−1)	High (−1)	No (-)	No (-)	Low (-)	−1.38 (−2.2, −0.35)	Low
Commissions	4 (8)	High (−1)	Null	No (-)	No (-)	Low (-)	−0.62 (−1.01, −0.23)	Moderate
Correct hits	4 (7)	High (−1)	High (−1)	No (-)	No (-)	Low (-)	−1.50 (−2.53, −0.38)	Low
Reaction time	4 (5)	High (−1)	Low	No (-)	No (-)	Low (-)	−0.67 (−1.27, −0.07)	Moderate
Perceptual sensitivity	4 (5)	High (−1)	Moderate	No (-)	No (-)	Low (-)	−1.07 (−1.92, −0.22)	Moderate

Note: RCT: randomized control trial; CI: confidence interval; SMD: standardized mean difference; (−1) = downgrade one level; (-) = same level.

## Data Availability

The data presented in this study are available on request from the corresponding author.
